# First Complete Genome Sequences of *Staphylococcus aureus* subsp. *aureus* Rosenbach 1884 (DSM 20231^T^), Determined by PacBio Single-Molecule Real-Time Technology

**DOI:** 10.1128/genomeA.00800-15

**Published:** 2015-07-16

**Authors:** Akino Shiroma, Yasunobu Terabayashi, Kazuma Nakano, Makiko Shimoji, Hinako Tamotsu, Noriko Ashimine, Shun Ohki, Misuzu Shinzato, Kuniko Teruya, Kazuhito Satou, Takashi Hirano

**Affiliations:** Okinawa Institute of Advanced Sciences, Uruma, Okinawa, Japan

## Abstract

The first complete genome sequences of *Staphylococcus aureus* subsp. *aureus* Rosenbach 1884 strain DSM 20231^T^, the type strain of the bacterium causing staphylococcal disease, were determined using PacBio RS II. The sequences represent the chromosome (2,755,072 bp long; G+C content, 32.86%) and a plasmid (27,490 bp long; G+C content, 30.69%).

## GENOME ANNOUNCEMENT

*Staphylococcus aureus* is a Gram-positive, non-spore-forming, and nonmotile coccus. It is a human indigenous bacterium, and about 30% of healthy humans harbor it in their nasal passages (1). *S. aureus* is the major cause of staphylococcal disease. The emergence of antibiotic-resistant forms of pathogenic *S. aureus* is a worldwide problem in clinical medicine (2, 3). *S. aureus* has a genome size of approximately 2.8 Mb, with a G+C content of 32%. The chromosome contains pathogenicity islands and antibiotic resistance genes acquired through horizontal gene transfer of mobile genetic elements (MGEs) (4, 5). The MGEs occupy 15% to 20% of the chromosome (6, 7). During outbreaks, *S. aureus* genomes develop single nucleotide polymorphisms (SNPs) and small genetic rearrangements and acquire or lose MGEs containing resistance or virulence genes (8–10).

The type strain of *S. aureus* subsp. *aureus* Rosenbach 1884 (DSM 20231^T^) was first isolated in 1884 from human pleural fluid by Rosenbach (11). At present, >60 complete genome sequences of *S. aureus* are publicly available (http://www.ncbi.nlm.nih.gov/genome/genomes/154); however, the genome of the type strain of this species has not been completely sequenced (12). The type strain exhibits all of the relevant genotypic and phenotypic properties cited in the species circumscriptions; therefore, the complete genome sequence of the type strain is crucial for analyzing, comparing, and evaluating the characteristics of the species. Here, we report the first complete genome sequences of *S. aureus* subsp. *aureus* DSM 20231^T^ determined by the single-molecule real-time (SMRT) technology (13).

Genomic DNA obtained from Deutsche Sammlung von Mikroorganismen und Zellkulturen (DSMZ) was purified using the PowerClean DNA cleanup kit (Mo Bio Laboratories, Carlsbad, CA), followed by 20-kb library construction for P5-C3 chemistry without shearing. Eight SMRT cells of the libraries were sequenced using the PacBio RS II platform (Pacific Biosciences, Menlo Park, CA) with 180-min movies. The maximum and average subread lengths were 34,694 and 6,038 bp, respectively. *De novo* assembly was conducted by using the Hierarchical Genome Assembly Process 3 (HGAP3) workflow (14). Two circular contigs were obtained, one representing a chromosome (2,755,072 bp; average G +C content, 32.86%; maximum, 60%), and the other representing a plasmid (27,490 bp; G+C content, 30.69%). The chromosome included 29 sets of identical sequence pairs >1,000 bp in size (maximum, 3,063 bp) and several notable tandem repeats (e.g., 384 bp × 5 copies and 18 bp × 42 copies). The SMRT technology provides multikilobase extra-long reads and thereby can resolve these hard-to-sequence regions (15).

The complete genome sequences of the type strain of *S. aureus* subsp. *aureus* reported here can be used as the standard reference for the species and will accelerate the understanding of the pathogenomic characteristics of the species and their role in (antibiotic-resistant) staphylococcal disease.

### Nucleotide sequence accession numbers.

The complete genome sequences of the *S. aureus* subsp. *aureus* DSM 20231^T^ chromosome and plasmid were deposited in DDBJ/ENA/GenBank under the accession numbers CP011526 and CP011527, respectively.

## References

[1] Gorwitz RJ, Kruszon-Moran D, McAllister SK, McQuillan G, McDougal LK, Fosheim GE, Jensen BJ, Killgore G, Tenover FC, Kuehnert MJ 2008 Changes in the prevalence of nasal colonization with *Staphylococcus aureus* in the United States, 2001–2004. J Infect Dis 197:1226–1234. doi:10.1086/533494. 18422434

[2] Grundmann H, Aanensen DM, van den Wijngaard CC, Spratt BG, Harmsen D, Friedrich AW, European Staphylococcal Reference Laboratory Working Group 2010 Geographic distribution of *Staphylococcus aureus* causing invasive infections in Europe: a molecular-epidemiological analysis. PLoS Med 7:e1000215. doi:10.1371/journal.pmed.1000215. 20084094 PMC2796391

[3] Holden MT, Hsu LY, Kurt K, Weinert LA, Mather AE, Harris SR, Strommenger B, Layer F, Witte W, de Lencastre H, Skov R, Westh H, Zemlicková H, Coombs G, Kearns AM, Hill RL, Edgeworth J, Gould I, Gant V, Cooke J, Edwards GF, McAdam PR, Templeton KE, McCann A, Zhou Z, Castillo-Ramírez S, Feil EJ, Hudson LO, Enright MC, Balloux F, Aanensen DM, Spratt BG, Fitzgerald JR, Parkhill J, Achtman M, Bentley SD, Nübel U 2013 A genomic portrait of the emergence, evolution, and global spread of a methicillin-resistant *Staphylococcus aureus* pandemic. Genome Res 23:653–664. doi:10.1101/gr.147710.112. 23299977 PMC3613582

[4] Ito T, Katayama Y, Hiramatsu K 1999 Cloning and nucleotide sequence determination of the entire *mec* DNA of pre-methicillin-resistant *Staphylococcus aureus* N315. Antimicrob Agents Chemother 43:1449–1458. 10348769 10.1128/aac.43.6.1449 PMC89295

[5] Katayama Y, Ito T, Hiramatsu K 2000 A new class of genetic element, Staphylococcus cassette chromosome *mec*, encodes methicillin resistance in *Staphylococcus aureus*. Antimicrob Agents Chemother 44:1549–1555. doi:10.1128/AAC.44.6.1549-1555.2000. 10817707 PMC89911

[6] Lindsay JA, Holden MT 2004 *Staphylococcus aureus*: superbug, super genome? Trends Microbiol 12:378–385. doi:10.1016/j.tim.2004.06.004. 15276614

[7] Lindsay JA, Moore CE, Day NP, Peacock SJ, Witney AA, Stabler RA, Husain SE, Butcher PD, Hinds J 2006 Microarrays reveal that each of the ten dominant lineages of *Staphylococcus aureus* has a unique combination of surface-associated and regulatory genes. J Bacteriol 188:669–676. doi:10.1128/JB.188.2.669-676.2006. 16385056 PMC1347281

[8] Kuroda M, Ohta T, Uchiyama I, Baba T, Yuzawa H, Kobayashi I, Cui L, Oguchi A, Aoki K, Nagai Y, Lian J, Ito T, Kanamori M, Matsumaru H, Maruyama A, Murakami H, Hosoyama A, Mizutani-Ui Y, Takahashi NK, Sawano T, Inoue R, Kaito C, Sekimizu K, Hirakawa H, Kuhara S, Goto S, Yabuzaki J, Kanehisa M, Yamashita A, Oshima K, Furuya K, Yoshino C, Shiba T, Hattori M, Ogasawara N, Hayashi H, Hiramatsu K 2001 Whole genome sequencing of meticillin-resistant *Staphylococcus aureus*. Lancet 357:1225–1240. doi:10.1016/S0140-6736(00)04403-2. 11418146

[9] Holden MT, Feil EJ, Lindsay JA, Peacock SJ, Day NP, Enright MC, Foster TJ, Moore CE, Hurst L, Atkin R, Barron A, Bason N, Bentley SD, Chillingworth C, Chillingworth T, Churcher C, Clark L, Corton C, Cronin A, Doggett J, Dowd L, Feltwell T, Hance Z, Harris B, Hauser H, Holroyd S, Jagels K, James KD, Lennard N, Line A, Mayes R, Moule S, Mungall K, Ormond D, Quail MA, Rabbinowitsch E, Rutherford K, Sanders M, Sharp S, Simmonds M, Stevens K, Whitehead S, Barrell BG, Spratt BG, Parkhill J 2004 Complete genomes of two clinical *Staphylococcus aureus* strains: evidence for the rapid evolution of virulence and drug resistance. Proc Natl Acad Sci U S A 101:9786–9791. doi:10.1073/pnas.0402521101. 15213324 PMC470752

[10] Lindsay JA 2014 Evolution of *Staphylococcus aureus* and MRSA during outbreaks. Infect Genet Evol 21:548–553. doi:10.1016/j.meegid.2013.04.017. 23665384

[11] Cowan ST, Shaw C, Williams RE 1954 Type strain for *Staphylococcus aureus* Rosenbach. J Gen Microbiol 10:174–176. doi:10.1099/00221287-10-1-174. 13130839

[12] Kim BS, Yi H, Chun J, Cha CJ 2014 Genome sequence of type strain of *Staphylococcus aureus* subsp. *aureus*. Gut Pathog 6:6. doi:10.1186/1757-4749-6-6. 24628867 PMC3985588

[13] Eid J, Fehr A, Gray J, Luong K, Lyle J, Otto G, Peluso P, Rank D, Baybayan P, Bettman B, Bibillo A, Bjornson K, Chaudhuri B, Christians F, Cicero R, Clark S, Dalal R, Dewinter A, Dixon J, Foquet M, Gaertner A, Hardenbol P, Heiner C, Hester K, Holden D, Kearns G, Kong X, Kuse R, Lacroix Y, Lin S, Lundquist P, Ma C, Marks P, Maxham M, Murphy D, Park I, Pham T, Phillips M, Roy J, Sebra R, Shen G, Sorenson J, Tomaney A, Travers K, Trulson M, Vieceli J, Wegener J, Wu D, Yang A, Zaccarin D, Zhao P, Zhong F, Korlach J, Turner S 2009 Real-time DNA sequencing from single polymerase molecules. Science 323:133–138. doi:10.1126/science.1162986. 19023044

[14] Chin CS, Alexander DH, Marks P, Klammer AA, Drake J, Heiner C, Clum A, Copeland A, Huddleston J, Eichler EE, Turner SW, Korlach J 2013 Nonhybrid, finished microbial genome assemblies from long-read SMRT sequencing data. Nat Methods 10:563–569. doi:10.1038/nmeth.2474. 23644548

[15] Koren S, Harhay GP, Smith TP, Bono JL, Harhay DM, Mcvey SD, Radune D, Bergman NH, Phillippy AM 2013 Reducing assembly complexity of microbial genomes with single-molecule sequencing. Genome Biol 14:R101. doi:10.1186/gb-2013-14-9-r101. 24034426 PMC4053942

